# Role of Diffusion Tensor Imaging-Derived Metrics for the Assessment of Deranged Myelination in Children With Developmental Delay: A Hospital-Based Observational Study

**DOI:** 10.7759/cureus.96972

**Published:** 2025-11-16

**Authors:** Swathi Karumanchi, Harish Marvada, G Murugan, Ashwin Kumar

**Affiliations:** 1 Radiodiagnosis, Sree Balaji Medical College and Hospital, Chennai, IND; 2 Radiology, Sree Balaji Medical College and Hospital, Chennai, IND; 3 Radiodiagnosis, Barnard Institute of Radiology, Madras Medical College, Chennai, IND

**Keywords:** diffusion tensor imaging, fractional anisotropy, neurodevelopment, radial diffusivity, white matter

## Abstract

Background

Diffusion tensor imaging (DTI) is a sensitive neuroimaging modality that evaluates white matter microstructure by measuring parameters such as fractional anisotropy (FA), mean diffusivity (MD), radial diffusivity (RD), and axial diffusivity (AD). Alterations in these metrics can indicate myelin disruption, axonal injury, or generalized microstructural changes. This study aimed to assess white matter integrity in children with developmental delay and evaluate the diagnostic performance of DTI metrics using receiver operating characteristic (ROC) analysis.

Methods

A hospital-based observational study was conducted on pediatric subjects with neurodevelopmental abnormalities. DTI was performed, and FA, MD, RD, and AD were quantified across major tracts, including the genu and splenium of the corpus callosum, anterior and posterior limbs of the internal capsule, superior longitudinal fasciculus (SLF), inferior fronto-occipital fasciculus (IFOF), and frontal and parietal white matter. Age-matched controls served as the comparison group. Statistical analysis included group comparisons and ROC curve evaluation for diagnostic accuracy.

Results

Significant reductions in FA were observed in the genu of the corpus callosum (0.34±0.07), frontal white matter (0.31±0.06), parietal white matter (0.33±0.05), SLF (0.35±0.06), and IFOF (0.37±0.05) (p<0.01 vs. controls). RD was significantly elevated in these regions (0.92-0.97×10⁻³ mm²/s; p<0.01), consistent with demyelination or delayed myelination. MD values were diffusely elevated (1.06-1.12×10⁻³ mm²/s), supporting generalized microstructural disruption, whereas AD showed mild but non-significant changes. ROC analysis demonstrated that FA had the highest diagnostic accuracy: genu of the corpus callosum (AUC 0.89), frontal white matter (AUC 0.91), and SLF (AUC 0.88). RD and MD also showed strong discriminatory ability, while AD performed less robustly.

Conclusion

Reduced FA, along with elevated RD and MD, reliably reflects white matter microstructural injury in pediatric populations. ROC analysis confirmed FA as the most sensitive biomarker, with high sensitivity and specificity. DTI metrics hold strong clinical potential for the early detection of neurodevelopmental white matter abnormalities.

## Introduction

Diffusion tensor imaging (DTI) is an advanced magnetic resonance imaging (MRI) technique that has revolutionized the in vivo examination of the brain's white matter microstructure. Unlike conventional MRI, which provides superb anatomical detail of gray matter and gross white matter structures, DTI is sensitive to the microscopic movement of water molecules within biological tissues. This technique allows for the visualization and quantification of the diffusion of water, which is not random in the brain but is constrained and directed by the organization of cellular structures. Within the tightly packed axons of white matter tracts, water molecules diffuse more freely along the direction of the fibers than perpendicular to them, a property known as anisotropic diffusion. By measuring this directionality, DTI provides a unique window into the structural integrity, organization, and connectivity of neural pathways [1].

The core principle of DTI involves quantifying the magnitude and direction of water diffusion in three-dimensional space. This is mathematically represented by a tensor, from which several key quantitative metrics are derived. Fractional anisotropy (FA) is the most commonly used metric, representing the degree of directionality of water diffusion. FA values range from 0 (perfectly isotropic diffusion, as in cerebrospinal fluid) to 1 (perfectly anisotropic diffusion, indicative of highly coherent and intact fiber tracts). Mean diffusivity (MD) reflects the overall magnitude of water diffusion, irrespective of direction; elevated MD often indicates increased extracellular space due to edema or neuronal loss. Furthermore, DTI allows for the decomposition of diffusion into axial diffusivity (AD), which measures diffusion parallel to the axonal fibers, and radial diffusivity (RD), which measures diffusion perpendicular to the fibers. Changes in AD and RD are thought to provide more specific insights into underlying pathology, with decreased AD potentially suggesting axonal damage and increased RD being strongly associated with demyelination [2].

The clinical and research utility of DTI is vast. In neurodegenerative conditions like Alzheimer's and Parkinson's disease, DTI can detect subtle microstructural changes in white matter that precede overt atrophy visible on conventional MRI [3]. In multiple sclerosis, it is instrumental in characterizing the dual pathological processes of demyelination (reflected by increased RD) and axonal transection (reflected by decreased AD). DTI also plays a crucial role in the evaluation of stroke, traumatic brain injury, and brain tumors, where it can delineate the degree of white matter infiltration and displacement, thereby guiding surgical planning and therapeutic interventions [4]. A powerful application of DTI is tractography, a post-processing technique that reconstructs the three-dimensional pathways of major white matter tracts. This is particularly valuable in pre-surgical planning for lesions near eloquent pathways, helping to minimize postoperative neurological deficits [5,6].

A critical area where DTI shows significant promise is in the evaluation of pediatric brain disorders, particularly developmental delay. Developmental delay is a common clinical concern, affecting an estimated 10-15% of children, and encompasses a spectrum of conditions characterized by a significant lag in reaching milestones in motor, cognitive, communication, or social skills [7]. The etiologies of developmental delay are exceedingly heterogeneous, ranging from genetic syndromes and chromosomal abnormalities to prenatal insults such as infections, toxins, and hypoxic-ischemic events. Despite this diversity in causation, the clinical presentations can be similar, often necessitating intensive and prolonged rehabilitation therapies to harness brain plasticity. A fundamental challenge in managing developmental delay is that conventional MRI, while a crucial component of the diagnostic workup, is frequently reported as "normal" in a substantial proportion of cases [8]. This absence of a clear structural correlate can complicate clinical communication and hinder the justification for long-term therapeutic commitments.

This is where DTI offers a paradigm shift. The technique is exquisitely sensitive to the microstructural changes that accompany brain maturation. During normal development, the brain undergoes rapid organizational changes, including axonal growth, myelination, and synaptic pruning, all of which influence water diffusion. There is a well-established trajectory of increasing FA and decreasing MD in white matter tracts as the brain matures from infancy through adolescence [9]. Consequently, deviations from these typical developmental patterns can serve as biomarkers for aberrant neurodevelopment. Recent DTI studies have consistently demonstrated widespread alterations in white matter microstructure in children with various developmental disorders, including autism spectrum disorder and cerebral palsy, even when conventional MRI appears unremarkable [4,10]. These findings suggest that DTI can reveal a "hidden pathology" at the microstructural level.

Therefore, DTI-derived metrics, such as FA, MD, AD, and RD, have the potential to serve as non-invasive, quantitative neuroimaging proxies for assessing the integrity of cerebral white matter in children with developmental delay. By providing objective, numerical data, DTI can move the evaluation beyond the subjective interpretation of "normal" or "abnormal" on conventional scans. This study aims to test the hypothesis that children with a clinical diagnosis of developmental delay, but with normal findings on routine MRI, exhibit significant alterations in DTI metrics within predefined white matter regions compared to typically developing children. The primary objective is to evaluate the role of DTI in identifying deranged myelination and axonal integrity in this patient population, thereby providing statistical and quantitative validation for its use as a sensitive biomarker in the diagnostic and prognostic assessment of developmental delay.

## Materials and methods

Study setting

This research was conducted in the Department of Radiodiagnosis at Sree Balaji Medical College and Hospital, Chennai, India. The institution is equipped with advanced imaging facilities, including a 3 Tesla MRI scanner, which made it an appropriate setting for the study.

Study design and duration

The present study was designed as an observational cross-sectional study, spanning a period of 24 months from July 2023 to June 2025. The design was chosen to capture detailed cross-sectional data on children with suspected developmental delay and to compare them with age-matched controls who underwent MRI for other indications but had normal findings.

Study population and sample size

The study population comprised children with a clinical suspicion of developmental delay, along with children referred to the radiology department for other medical indications who demonstrated normal findings on routine MRI. To calculate the required sample size, with a 90% confidence level, corresponding to a Z value of 1.65, a variance effect of 25%, and a margin of error of 10%, the calculated sample size was 30. 

Inclusion and exclusion criteria

Children between the ages of two and 15 years of either gender were considered eligible if they were clinically suspected of having a developmental delay. Only those whose parents or guardians provided informed consent were included. Exclusion criteria were stringent to eliminate confounding factors. Children with visible morphological abnormalities on standard MRI sequences were excluded, as were those with a history or presence of craniospinal infections, systemic diseases, or chronic medical conditions that might interfere with imaging outcomes. Additional exclusions included children with metallic implants or pacemakers, those suffering from claustrophobia, and cases in which parental consent was not granted. In this study, children with known medical conditions that could independently contribute to structural neurological changes, such as preterm birth, perinatal asphyxia, head injury, central nervous system infections, or known genetic/neurocutaneous syndromes, were excluded. Thus, the participants with developmental delay were those without identifiable prior neurological insults, presenting with developmental delay of uncertain origin.

Ethical considerations

Prior to commencement, ethical clearance was obtained from the Institutional Human Ethics Committee of Sree Balaji Medical College and Hospital (approval number: 002/SBMC/IHEC/2023/1950). Informed consent was sought from parents or guardians of all participants after explaining the objectives and procedures of the study in a language they understood.

Data collection procedure

The methodology involved a stepwise approach beginning with the identification of eligible participants based on clinical suspicion of developmental delay. Following eligibility assessment and consent, children underwent MRI combined with DTI. Data acquisition was performed on a 3 Tesla GE Signa Pioneer MRI scanner (GE Healthcare, Chicago, Illinois, United States). DTI data were processed to generate quantitative metrics, namely, FA, MD, AD, and RD. These parameters were used to assess subtle microstructural alterations in the brain's white matter. Additionally, metabolite ratios were studied and correlated with the clinical diagnosis of developmental delay. Receiver operating characteristic (ROC) analysis was applied to determine optimal cut-off values for these parameters, thus establishing thresholds with maximum sensitivity and specificity for differentiating normal development from developmental delay.

Imaging protocols

All imaging was carried out on a 3 Tesla GE Signa Pioneer scanner. Participants were placed in the supine position, head first, with their heads stabilized using cushions and aligned with a laser beam localizer centered at the glabella. A head and neck 64-channel coil was used for image acquisition.

Standard imaging sequences included the following: T1-weighted axial sequence (TR 2000 ms, TE 9 ms, slice thickness 4 mm), T2-weighted axial sequence (TR 5000 ms, TE 100 ms, slice thickness 4 mm), fluid-attenuated inversion recovery (FLAIR) axial sequence (TR 9000 ms, TE 80 ms, slice thickness 4 mm), T2-weighted coronal sequence (TR 5000 ms, TE 100 ms, slice thickness 4 mm), T1-weighted sagittal sequence (TR 2000 ms, TE 9 ms, slice thickness 4 mm), and diffusion-weighted imaging (DWI) sequence (TR 5000 ms, TE 60 ms, slice thickness 4 mm, b-value 800 s/mm²).

The principal DTI sequence employed was "ep2d_diff_mddw_20_(DTI)", which applies multiple diffusion gradients in 20 distinct directions using an echo planar imaging (EPI) technique. The acquisition parameters for DTI were as follows: TR 3700 ms, TE 92 ms, b-values of 0 and 1000 seconds/mm², voxel size of 1.7×1.7×4 mm, and a slice thickness of 4 mm with a 30% interslice distance factor. Twenty-five slices without interslice gaps were obtained to comprehensively include the cerebellum, brainstem, and cerebral hemispheres. The total acquisition time for the diffusion-weighted sequence was approximately four minutes and 39 seconds. The data were processed using the Syngo Via XA 11 software (Siemens Healthineers, Erlangen, Germany), with quantitative analysis performed through the manual placement of regions of interest (ROIs) on the generated parametric maps. The regions selected corresponded to major white matter pathways originating from functionally significant brain regions.

Measurements and derived parameters

DTI works on the principle of measuring water molecule motion within tissues. By modeling water diffusion as a tensor, DTI produces a set of scalar and vector data describing the directional diffusion properties within each voxel. Among the derived parameters, FA serves as the most significant metric. It quantifies the directionality of water diffusion and reflects the structural integrity and organization of white matter tracts. In developing brains, FA changes precede alterations detectable on conventional T1- or T2-weighted MRI, making it an earlier and more sensitive indicator of white matter maturation. Other parameters include MD, which measures the average magnitude of water diffusion; AD, which quantifies diffusion along the main axis of a fiber tract; and RD, which measures diffusion perpendicular to the fiber tracts. Together, these metrics provide insights into both axonal integrity and myelination status.

Tractography

In addition to scalar measurements, tractography was employed as a three-dimensional modeling technique to visualize the orientation of white matter tracts. Fiber tracking was initiated at seed voxels and propagated based on the principal eigenvectors of adjacent voxels, representing the major axes of diffusion ellipsoids. Tracking continued in both directions until the FA fell below a pre-set threshold, at which point tract extension was terminated. This approach allowed for the reconstruction of continuous neural pathways, revealing areas of tract reduction or loss that are characteristic of developmental delay.

Image analysis

The analysis focused on identifying deviations in FA and other DTI parameters between children with developmental delay and controls with normal MRI findings. Special attention was paid to key white matter regions such as the corpus callosum, internal capsule, and corticospinal tracts. These were chosen due to their critical roles in motor and cognitive development. Any observed reduction in FA or abnormality in MD, AD, or RD values was interpreted as evidence of microstructural alteration.

Statistical analysis

The collected data were entered into Microsoft Excel 2010 (Microsoft Corporation, Redmond, Washington, United States) and further analyzed using Epi Info Version 7.2.0 (Centers for Disease Control and Prevention, Atlanta, Georgia, United States). Descriptive statistics were employed to summarize demographic and clinical variables, with continuous data expressed as mean±standard deviation (SD) and categorical data presented as frequencies and percentages. Inferential statistics were applied to test the significance of observed differences between groups. Student's t-test was used to compare continuous variables, while categorical data were assessed using chi-squared tests where appropriate. The diagnostic performance of DTI metrics was evaluated through ROC analysis, with the area under the curve (AUC) calculated to determine the discriminative ability of FA and other diffusivity measures. Optimal cut-off points providing the best balance of sensitivity and specificity were identified, thereby supporting the clinical utility of DTI in detecting subtle abnormalities in children with developmental delay.

## Results

Out of the total 30 participants, the study population was between one and 12 years old, with an average age of 6.1±1.2 years. The cohort included 18 males (60%) and 12 females (40%), resulting in a male-to-female ratio of 1.5 to 1 (Table 1).

**Table 1:** Age and gender distribution of the study population (n=30)

Gender	Frequency	Percentage
Age (in years) (mean±SD)	6.1±1.2 years	
Male	18	60
Female	12	40
Total	30	100

**Figure 1:**
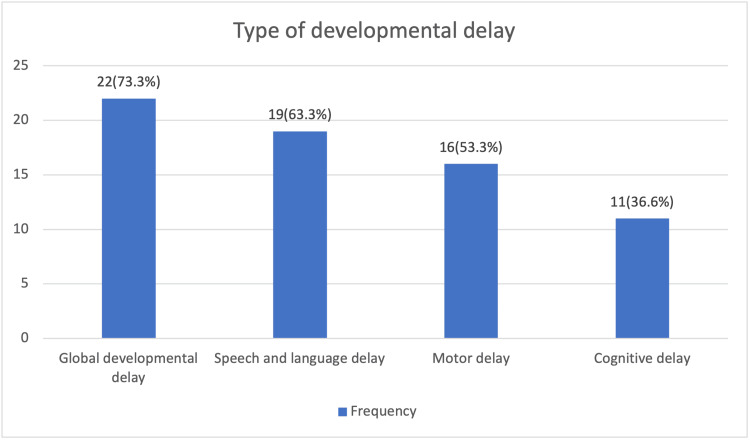
Distribution of the type of developmental delay among the study participants (n=30) This image shows the distribution of the type of developmental delay among the study participants in n(%) with the type being classified.

The most common developmental delay observed was global developmental delay in 22 (73.33%), followed by speech and language delay in 19 (63.33%), motor delay in 16 (53.3%), and cognitive delay in 11 (36.66%). Many patients presented with multiple overlapping delays (Figure 1).

In the present study, DTI parameters demonstrated notable microstructural alterations in children with developmental delay compared to normal controls in Table 2. FA values were significantly reduced in the genu of the corpus callosum (0.34±0.07), fronto-parietal white matter (0.31±0.06), superior longitudinal fasciculus (SLF) (0.35±0.06), and inferior fronto-occipital fasciculus (IFOF) (0.37±0.05) (p<0.01). Concurrently, RD was markedly elevated in these regions, with values such as 0.93±0.10 in the genu, 0.97±0.11 in frontal white matter, and 0.94±0.11 in the SLF (p<0.01), strongly indicating impaired or delayed myelination. MD was also elevated in affected regions, including 1.12±0.14 in frontal white matter and 1.09±0.13 in parietal white matter, reflecting generalized disruption of white matter microstructure. AD values, though slightly elevated (e.g., 1.25±0.13 in the genu, 1.26±0.13 in frontal white matter), were not statistically significant. 

**Table 2:** DTI metrics across major white matter tracts in children with developmental delay FA: fractional anisotropy; MD: mean diffusivity; RD: radial diffusivity; AD: axial diffusivity; IC: internal capsule; SLF: superior longitudinal fasciculus; IFOF: inferior fronto-occipital fasciculus; DTI: diffusion tensor imaging

Region	FA (mean±SD)	MD (×10⁻³ mm²/s)	RD	AD	Interpretation
Genu of the corpus callosum	0.34±0.07	1.06±0.11	0.93±0.10	1.25±0.13	↓FA, ↑RD suggest demyelination
Splenium	0.46±0.06	0.95±0.09	0.80±0.08	1.12±0.10	Mild FA reduction
Anterior limb of IC	0.40±0.05	1.02±0.12	0.87±0.10	1.18±0.12	↓FA, ↑RD
Posterior limb of IC	0.58±0.04	0.80±0.08	0.65±0.07	1.02±0.09	Near-normal
Frontal white matter	0.31±0.06	1.12±0.14	0.97±0.11	1.26±0.13	Significant microstructural damage
Parietal white matter	0.33±0.05	1.09±0.13	0.92±0.10	1.23±0.12	Altered integrity
SLF	0.35±0.06	1.10±0.13	0.94±0.11	1.22±0.12	↓FA, ↑RD
IFOF	0.37±0.05	1.08±0.12	0.91±0.10	1.20±0.12	Disrupted tract microstructure

Incomplete or attenuated tracts were visualized in 21 (70%) cases in frontal association fibers (SLF and IFOF). Corpus callosum thinning was evident in tractographic views in 18 (60%) children, especially in the genu. Corticospinal tracts appeared largely preserved in 24( 80%) subjects. Lower FA values in the frontal white matter and SLF were significantly correlated with the severity of language delay (r=-0.59; p=0.002). Higher RD values in parietal white matter were correlated with motor delay scores (r=0.54; p=0.005). Children having global developmental delay had the most extensive microstructural abnormalities across multiple tracts.

**Figure 2:**
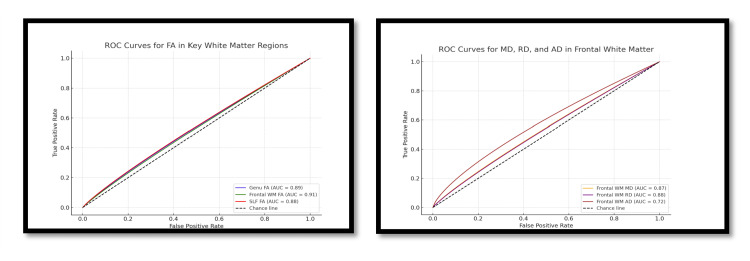
ROC of FA in key white matter and MD, RD, and AD of white matter regions The ROC analysis of DTI metrics across key white matter tracts demonstrated strong diagnostic performance in differentiating children with developmental delay from controls as shown in this table. ROC: receiver operating characteristic; FA: fractional anisotropy; MD: mean diffusivity; RD: radial diffusivity; AD: axial diffusivity; DTI: diffusion tensor imaging

The ROC analysis of DTI metrics across key white matter tracts demonstrated strong diagnostic performance in differentiating children with developmental delay from controls, as shown in Table 3. In the genu of the corpus callosum, FA showed an AUC of 0.89 with an optimal cut-off of <0.37 (sensitivity 87%; specificity 83%), while RD also performed well (AUC 0.86; cut-off >0.88×10⁻³ mm²/s). Similar findings were observed in the frontal white matter, where FA yielded the highest diagnostic accuracy (AUC 0.91; cut-off <0.33; sensitivity 90%; specificity 85%), supported by elevated MD and RD values with good sensitivity and specificity. The SLF also showed significant discriminatory ability, with FA (AUC 0.88; cut-off <0.36; sensitivity 86%; specificity 80%) and RD (AUC 0.85; cut-off >0.92×10⁻³ mm²/s; sensitivity 82%; specificity 78%) emerging as reliable indicators. Across regions, FA and RD consistently demonstrated superior performance, highlighting their value as sensitive markers of white matter microstructural abnormalities in developmental delay (Figure 2).

**Table 3:** ROC analysis of DTI metrics in the genu of corpus callosum, frontal white matter, and SLF Chi-squared test; p<0.05 is considered statistically significant AUC: area under the curve; FA: fractional anisotropy; MD: mean diffusivity; RD: radial diffusivity; AD: axial diffusivity; SLF: superior longitudinal fasciculus; ROC: receiver operating characteristic; DTI: diffusion tensor imaging

Region/metric	AUC (95% CI)	Cut-off value	Sensitivity	Specificity	χ² value	P-value
Genu of the corpus callosum						
FA	0.89 (0.78-0.97)	<0.37	87%	83%	14.6	<0.001
MD	0.84 (0.70-0.94)	>1.00×10⁻³ mm²/s	80%	77%	12.9	<0.001
RD	0.86 (0.74-0.95)	>0.88×10⁻³ mm²/s	83%	80%	13.7	<0.001
AD	0.71 (0.58-0.86)	>1.18×10⁻³ mm²/s	68%	70%	6.5	0.011
Frontal white matter						
FA	0.91 (0.82-0.98)	<0.33	90%	85%	16.8	<0.001
MD	0.87 (0.76-0.95)	>1.08×10⁻³ mm²/s	83%	80%	13.2	<0.001
RD	0.88 (0.77-0.96)	>0.95×10⁻³ mm²/s	85%	83%	14.1	<0.001
AD	0.72 (0.60-0.85)	>1.20×10⁻³ mm²/s	70%	65%	6.8	0.009
SLF						
FA	0.88 (0.75-0.96)	<0.36	86%	80%	13.9	<0.001
MD	0.83 (0.71-0.93)	>1.07×10⁻³ mm²/s	78%	75%	11.8	<0.001
RD	0.85 (0.73-0.94)	>0.92×10⁻³ mm²/s	82%	78%	12.5	<0.001
AD	0.70 (0.57-0.83)	>1.18×10⁻³ mm²/s	65%	67%	5.9	0.015

The Appendices section includes multiple DTI color-coded maps and tractography images. Color-coded DTI scans display ROIs in the anterior and posterior limbs of the bilateral internal capsule, as well as the genu and splenium of the corpus callosum. DTI measurements revealed reduced FA and elevated MD values in both the genu and splenium of the corpus callosum and reduced FA values in the anterior and posterior limbs of the bilateral internal capsule, with elevated MD values particularly in the anterior limbs. The DTI apparent diffusion coefficient (ADC) map further highlights these ROIs, confirming reduced FA and increased MD values in the anterior limbs of the internal capsules. In addition, the ROIs in the bilateral forceps minor demonstrated reduced FA in the right forceps minor, along with elevated MD values. Coronal and sagittal DTI tractography images show no significant decrease in the density of the corticospinal tracts or other major white matter fibers. DTI demonstrated the SLF bilaterally and the inferior longitudinal fasciculus on the left side. 

## Discussion

Developmental delay is a common neurodevelopmental disorder that presents a diagnostic challenge because of its heterogeneous etiology and varied clinical manifestations. Despite widespread reliance on conventional MRI, many children with clinically evident developmental delay show structurally normal scans, leaving physicians and families uncertain about prognosis and management. In this context, DTI provides a more sensitive method to assess microstructural white matter integrity, as it captures subtle changes in water diffusivity patterns that are not visible on routine MRI sequences. The present study explored whether DTI-derived quantitative metrics could differentiate children with developmental delay from controls, even in the absence of visible abnormalities on standard MRI, focusing particularly on the genu of the corpus callosum, frontal white matter, and SLF.

FA as a sensitive marker

One of the key findings of this study was the consistent reduction in FA values across all three major white matter regions studied. FA reflects the degree of directionality of water diffusion within axonal tracts, serving as a composite marker of axonal density, myelination, and overall fiber coherence. In our cohort, frontal white matter demonstrated the strongest diagnostic ability, with an AUC of 0.91 and a cut-off FA value of <0.33, yielding 90% sensitivity and 85% specificity. This finding is significant because the frontal lobes are among the last regions of the brain to myelinate and are crucial for executive functioning, attention, and working memory. Disruptions in their maturation may underlie many of the cognitive and behavioral impairments seen in developmental delay.

These results are in agreement with the work of Mukherjee et al., who found significant FA reductions in neonates with periventricular leukomalacia and later developmental impairments [11]. Similarly, Shashi et al. demonstrated altered FA values in children with 22q11.2 deletion syndrome, while Yoshida et al. reported reduced FA in autism spectrum disorders [12,13]. The consistency of FA alterations across various etiologies suggests that reduced FA is a robust transdiagnostic biomarker of abnormal neurodevelopment. Our study adds to this body of evidence by demonstrating that FA can serve as a reliable discriminator of developmental delay even when conventional imaging appears normal, with region-specific cut-off values enhancing clinical utility.

MD and white matter disorganization

MD was also found to be significantly elevated in the developmental delay group. It reflects the overall magnitude of water diffusion and is particularly sensitive to conditions where there is an expansion of extracellular space, loss of axonal density, or delayed myelination. In our study, MD changes were most pronounced in the frontal white matter, with an AUC of 0.87 and an optimal cut-off of >1.08×10⁻³ mm²/s, achieving 83% sensitivity and 80% specificity. These findings indicate generalized microstructural disorganization in the frontal regions, consistent with their role in higher-order cognitive functions. Wessam et al. previously reported elevated MD values in the corpus callosum and internal capsule of children with motor developmental delay [14]. Our results corroborate this observation while extending it to a broader cohort with clinically heterogeneous developmental delay. Furthermore, elevated MD across multiple tracts suggests that developmental delay is characterized by not only localized but also diffuse alterations in white matter organization.

RD and myelination deficits

RD emerged as another highly sensitive metric, with elevated values in all three key regions. It represents diffusion perpendicular to axonal fibers and is particularly sensitive to myelin integrity. In our cohort, the frontal white matter showed an AUC of 0.88, while the SLF and genu demonstrated AUCs of 0.85 and 0.83, respectively. The elevation of RD in the presence of reduced FA points strongly toward impaired or delayed myelination rather than primary axonal injury. This interpretation is supported by experimental work from Song et al., who demonstrated that RD is a reliable marker of dysmyelination in animal models, while AD remains relatively unaffected [15]. Our findings extend this principle to clinical populations, highlighting RD as a practical imaging biomarker for developmental delay. Importantly, the prominence of RD alterations underscores the hypothesis that developmental delay is primarily associated with maturational deficits in myelination rather than extensive axonal injury.

Limited diagnostic yield of AD

Unlike FA, MD, and RD, AD showed comparatively lower diagnostic value, with AUCs ranging between 0.70 and 0.72. While slightly elevated, AD did not differ significantly between groups, suggesting that axonal injury or degeneration is not the predominant pathophysiological mechanism in most children with developmental delay. This finding mirrors those of Kim et al. and Geng et al., who reported that AD alterations were less sensitive than FA or RD in predicting developmental outcomes in early childhood [16,17]. Taken together, these results suggest that FA and RD are more reliable for detecting subtle white matter abnormalities in children with normal-appearing structural MRIs.

Regional relevance and neuroanatomical correlations

The regional specificity of the findings provides important insights into the neuroanatomical underpinnings of developmental delay. The frontal white matter, genu of the corpus callosum, and SLF all demonstrated significant abnormalities, consistent with their known roles in executive functioning, interhemispheric communication, language, and spatial processing. These tracts mature progressively during infancy and early childhood, making them especially vulnerable to disruptions in myelination or axonal organization. The observed alterations thus provide a structural correlate for the heterogeneous cognitive and motor deficits seen in children with developmental delay.

The sample size in this study was relatively small (n=30), which may limit statistical power and generalizability. Larger, multicentric studies are needed to validate the derived cut-off values and improve external validity. The cross-sectional design also restricts the ability to assess developmental changes over time. Additionally, heterogeneity in age and clinical background among participants may have contributed to variability in diffusion metrics; future studies should consider age-stratified or more uniform cohorts. While DTI offers valuable insight into white matter microstructure, it is subject to motion artifacts, partial volume effects, and challenges in resolving crossing fibers. Advanced techniques such as high angular resolution diffusion imaging (HARDI) or diffusion spectrum imaging (DSI) may address these limitations. Moreover, the ROC analysis used to determine threshold values may be unstable in small samples and could overestimate diagnostic accuracy. Finally, correlation with standardized neuropsychological assessments was not performed but would strengthen structure-function interpretation in future research.

The clinical implications of these findings are considerable. First, DTI metrics can provide early diagnostic insights in children who have normal structural MRIs, allowing for the timely initiation of rehabilitation and cognitive therapies. Second, region-specific cut-off values may assist in subtyping developmental delay based on underlying white matter pathology, which can inform prognosis and therapeutic planning. Third, DTI can serve as a monitoring tool to evaluate treatment response and track neuroplastic changes over time, particularly in interventional studies. Finally, these metrics offer a valuable research tool for understanding the neurobiological basis of developmental disorders, potentially paving the way for biomarker-driven approaches to classification and management.

## Conclusions

This study demonstrates that DTI-derived metrics, particularly FA and RD, are highly sensitive indicators of microstructural white matter abnormalities in children with developmental delay who have normal conventional MRIs. The consistent reductions in FA and elevations in RD across key tracts, namely, frontal white matter, genu of the corpus callosum, and SLF, highlight delayed or impaired myelination as a predominant mechanism. By establishing region-specific cut-off values, this study provides a framework for integrating DTI into clinical workflows, enhancing early diagnosis, subtyping, and monitoring of developmental delay. Future research should focus on larger, longitudinal studies that incorporate neuropsychological assessments and advanced diffusion imaging techniques to refine our understanding of the structural basis of developmental disorders and translate imaging biomarkers into precision medicine approaches.

## Figures and Tables

**Figure 3:**
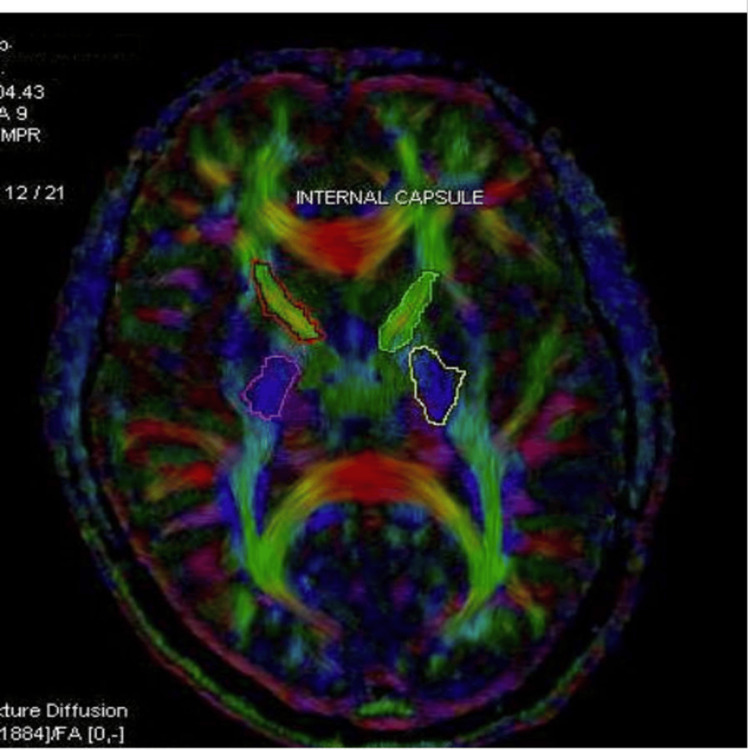
Image displaying a color-coded DTI scan with ROIs in anterior and posterior limbs of bilateral internal capsule DTI: diffusion tensor imaging; ROIs: regions of interest

**Figure 4:**
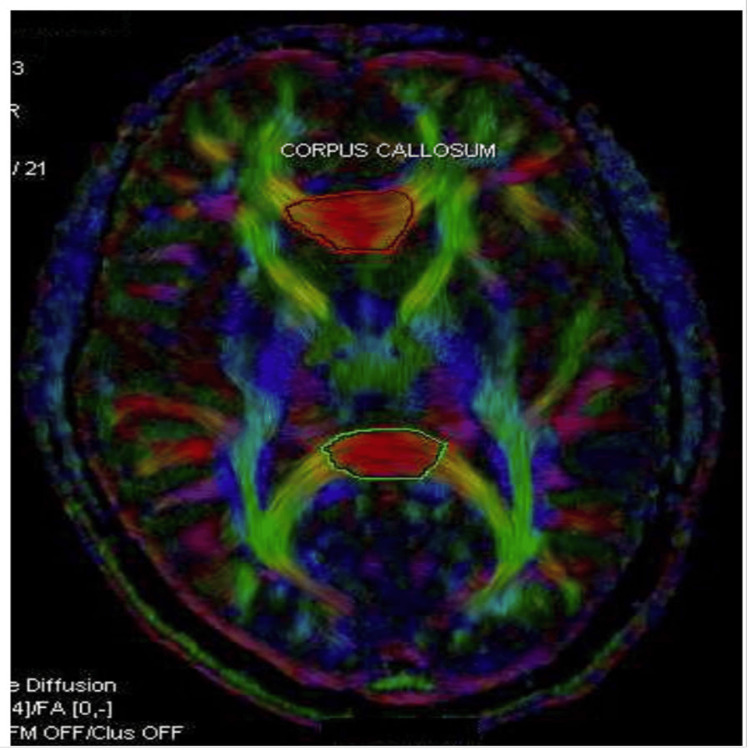
Image showing a color-coded DTI image highlighting the ROIs involving the genu and splenium of the corpus callosum DTI: diffusion tensor imaging; ROIs: regions of interest

**Figure 5:**
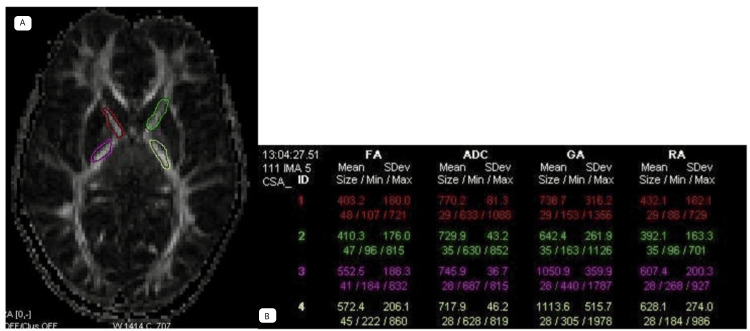
(A) The DTI ADC map illustrating ROIs in the bilateral internal capsule involving the anterior and posterior limbs. (B) DTI metrics indicating reduced FA values in both the anterior and posterior limbs of the bilateral internal capsule, along with elevated MD values observed at the anterior limbs of the bilateral internal capsule DTI: diffusion tensor imaging; ADC: apparent diffusion coefficient; ROIs: regions of interest; FA: fractional anisotropy; MD: mean diffusivity

**Figure 6:**
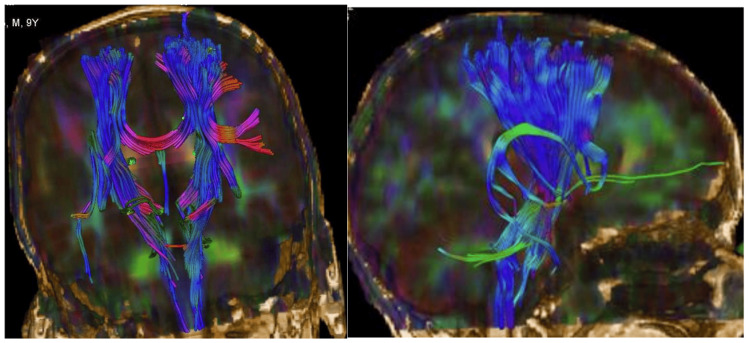
Coronal and sagittal DTI tractography images demonstrating no significant decrease in the density of the corticospinal tracts and other white matter fibers DTI: diffusion tensor imaging

**Figure 7:**
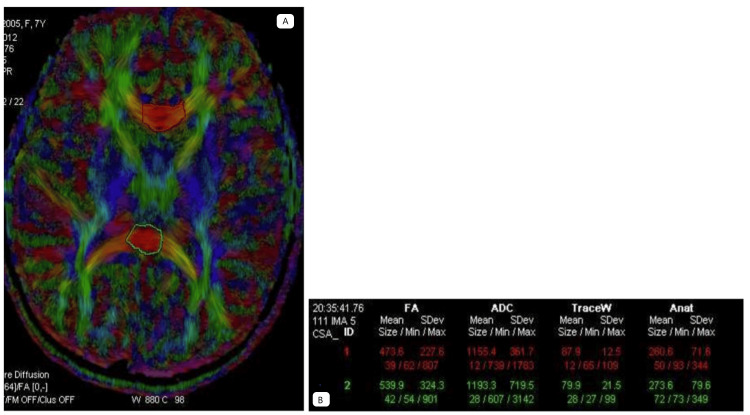
(A) The DTI color-coded map highlighting ROIs in the genu and splenium of the corpus callosum. (B) DTI measurements revealing a reduction in FA within the genu and elevated MD values in the splenium of the corpus callosum DTI: diffusion tensor imaging; ROIs: regions of interest; FA: fractional anisotropy; MD: mean diffusivity

**Figure 8:**
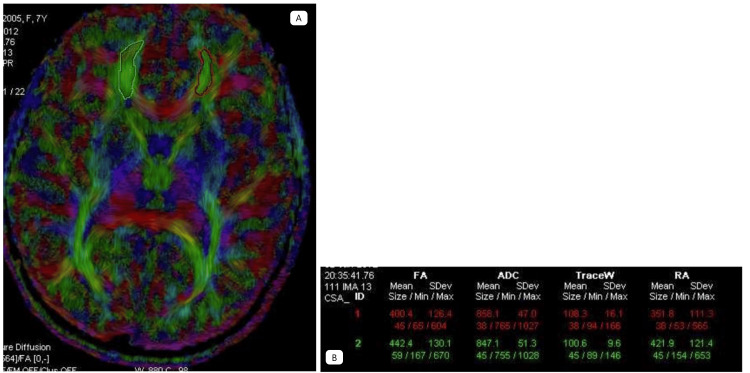
(A) The DTI color-coded map highlighting ROIs in the genu and splenium of the corpus callosum. (B) DTI measurements revealing a reduction in FA within the genu and elevated MD values in the splenium of the corpus callosum DTI: diffusion tensor imaging; ROIs: regions of interest; FA: fractional anisotropy; MD: mean diffusivity

**Figure 9:**
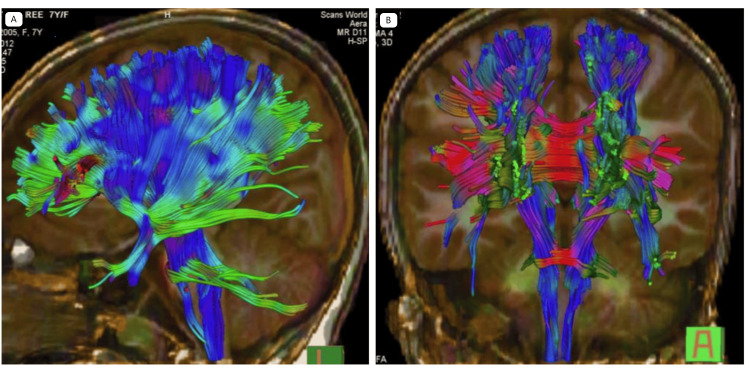
DTI tractography sagittal (A) and coronal (B) images revealing no notable decrease in the density of corticospinal tracts DTI: diffusion tensor imaging

**Figure 10:**
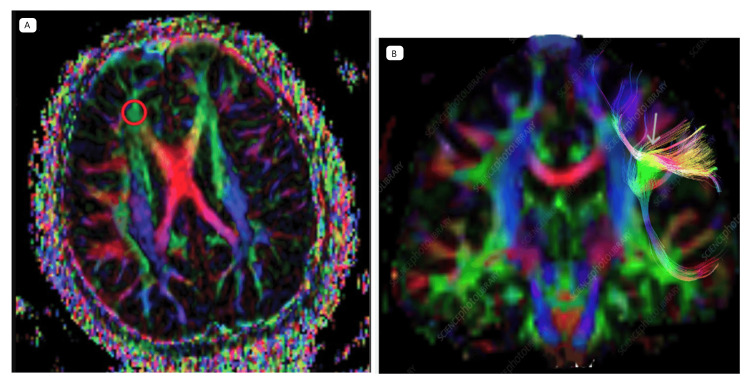
DTI showing superior longitudinal fasciculus on the right and left sides (A) DTI showing superior longitudinal fasciculus on the right side. (B) DTI fiber tractography of the superior longitudinal fasciculus on the left side DTI: diffusion tensor imaging

**Figure 11:**
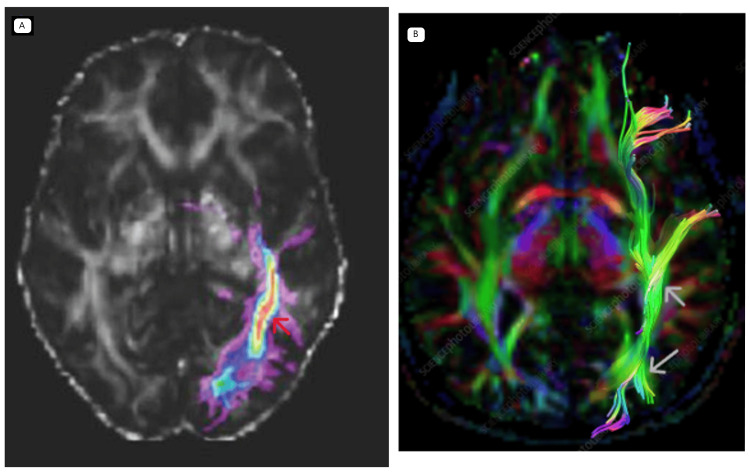
DTI showing left inferior longitudinal fasciculus (A) DTI showing left inferior longitudinal fasciculus. (B) DTI fiber tractography of the left inferior longitudinal fasciculus DTI: diffusion tensor imaging
